# Impact of Polystyrene Microplastics on Soil Properties, Microbial Diversity and *Solanum lycopersicum* L. Growth in Meadow Soils

**DOI:** 10.3390/plants14020256

**Published:** 2025-01-17

**Authors:** Shuming Liu, Yan Suo, Jinghuizi Wang, Binglin Chen, Kaili Wang, Xiaoyu Yang, Yaokun Zhu, Jiaxing Zhang, Mengchu Lu, Yunqing Liu

**Affiliations:** 1 Xinjiang Key Laboratory of Clean Conversion and High Value Utilization of Biomass Resources, Yili Normal University, Yining 835000, China; shumingl11@163.com (S.L.);; 2 School of Resources and Environment, Yili Normal University, Yining 835000, China

**Keywords:** plant growth, microbial diversity, nutrient cycling, soil properties, antioxidant enzyme

## Abstract

The pervasive presence of microplastics (MPs) in agroecosystems poses a significant threat to soil health and plant growth. This study investigates the effects of varying concentrations and sizes of polystyrene microplastics (PS-MPs) on the *Solanum lycopersicum* L.’s height, dry weight, antioxidant enzyme activities, soil physicochemical properties, and rhizosphere microbial communities. The results showed that the PS0510 treatment significantly increased plant height (93.70 cm, +40.83%) and dry weight (2.98 g, +100%). Additionally, antioxidant enzyme activities improved across treatments for *S. lycopersicum* L. roots. Physicochemical analyses revealed enhanced soil organic matter and nutrient levels, including ammonium nitrogen, phosphorus, and effective potassium. Using 16S rRNA sequencing and molecular ecological network techniques, we found that PS-MPs altered the structure and function of the rhizosphere microbial community associated with *S. lycopersicum* L. The PS1005 treatment notably increased microbial diversity and displayed the most complex ecological network, while PS1010 led to reduced network complexity and more negative interactions. Linear discriminant analysis effect size (LEfSe) analysis identified biomarkers at various taxonomic levels, reflecting the impact of PS-MPs on microbial community structure. Mantel tests indicated positive correlations between microbial diversity and soil antioxidant enzyme activity, as well as relationships between soil physicochemical properties and enzyme activity. Predictions of gene function revealed that PS-MP treatments modified carbon and nitrogen cycling pathways, with PS1005 enhancing methanogenesis genes (*mcrABG*) and PS1010 negatively affecting denitrification genes (*nirK*, *nirS*). This study provides evidence of the complex effects of PS-MPs on soil health and agroecosystem functioning, highlighting their potential to alter soil properties and microbial communities, thereby affecting plant growth.

## 1. Introduction

Polystyrene (PS) is a widely used plastic material, mainly used in packaging, construction materials, and other fields, accounting for 7.1% of the total global plastic production [1,2,3], and is also a significant contributor to microplastic pollution in the environment. The thickness of the PS film mulch is only 8–50 μm, and its low cost makes it widely used in agricultural production. However, the recycling rate of the film mulch is very low, resulting in its residue in the soil [4,5]. Residual PS film mulch is present in the soil environment in the form of particles, fragments, and fibers [6]. Prolonged exposure of PS films mulch to light can lead to structural damage, such as cracks and reticulation due to photodegradation, and ultimately the formation of PS-MPs with a particle size of less than 5 mm [7]. In addition, erosion and biofilm formation by soil microorganisms accelerate the decomposition of PS film mulch, further accelerating the production of PS-MPs [8]. Currently, PS-MP contamination in soil represents a significant environmental concern. Concentrations of MPs in agricultural soils range from 7100 to 42,900 particles per kilogram, with particle sizes predominantly between 0.05 and 1 mm [9]. Research indicates that greenhouse topsoil, particularly in the eastern coastal regions of China, exhibits exceptionally high levels of PS-MPs, reaching concentrations of up to 78.00 ± 12.91 particles per kg [10].

Meadow soils are characterized by their fertility and high organic matter content, typically ranging from 10% to 20%. These soils contain approximately 0.2% total nitrogen and around 0.075% total phosphorus. On average, crops can absorb over 250 kg of phosphorus per hectare through natural decomposition processes in these ecosystems [3]. However, plastic contamination can increase soil particle roughness and alter soil permeability, which may adversely affect water uptake by plants and root characteristics, thereby influencing plant growth [11]. Research indicates that certain MPs, such as high-density polyethylene, can lead to a decrease in soil pH [12]. Furthermore, MPs contamination alters soil pore size and enhances leaching, resulting in significant nitrogen loss, which disrupts the nitrogen cycle and negatively impacts nitrogen uptake by plants [12]. Additionally, MPs can reduce soil viscosity, increase soil porosity, interfere with the structure and function of soil microbial communities, accelerate nutrient loss, and negatively influence the rhizosphere environment [13,14]. Despite the significant impact of MPs on soil nutrients, few studies have been reported for meadow soils, so more in-depth research is needed.

*Solanum lycopersicum* L., is rich in vitamin A, vitamin C, vitamin E, potassium, magnesium, lycopene, and other nutrients, as well as some antioxidants [15,16]. Due to the unique natural conditions in the Xinjiang Uygur Autonomous Region, *S. lycopersicum* L. has become one of the major crops in the region. However, the growth and quality of *S. lycopersicum* L. can be affected by a variety of factors, such as the physicochemical properties of the soil, water management, light conditions, temperature variations, and fertilization practices [17]. Studies have shown that the content of nutrients, such as soil organic matter, available nitrogen, available phosphorus, and available potassium, have a significant effect on the growth, development, and yield of *S. lycopersicum* L. [18]. Sufficient nitrogen levels have been shown to enhance the nutritional quality of the *S. lycopersicum* L., particularly in increasing soluble sugars and vitamin C content [19]. In contemporary agricultural practices, film mulch technology is extensively employed in *S. lycopersicum* L. cultivation; the mulch enhances soil temperature and moisture levels, thus promoting the growth and development of *S. lycopersicum* L. One study reported that soil temperatures in mulched areas were elevated by 1.3 °C to 1.7 °C compared to unmulched areas, contributing positively to the growth of the *S. lycopersicum* L. [20]. However, the prolonged use of mulch may also lead to increased concentrations of MPs in the soil, resulting in contamination that can adversely affect soil health and agricultural productivity.

PS-MPs can accumulate in plant roots and subsequently transfer from the underground environment to the aboveground portions of the plant via the xylem and leaf veins [21]. The accumulation of PS-MPs within the root system impedes normal root development, resulting in reduced root length and weight [22]. This interference not only disrupts the growth of the root system but also negatively affects water uptake, leading to decreased chlorophyll content and impaired photosynthesis. Consequently, these factors inhibit overall plant growth and lead to reductions in biomass [23]. In pot experiments, the treatment of *Oryza sativa* L. with 1% PS-MPs (*w*/*w*) demonstrated significant adverse effects on plant growth, including a reduction in root length by 15–20%, a decrease in plant height by 15–30%, and a decline in fresh weight by 12–37% [24]. The oxidative stress caused by PS-MPs in plants was observed to be dependent on concentration; notably, lower levels of PS-MPs enhanced the activity of antioxidant enzymes in *Oryza sativa* L., while higher levels led to a reduction in antioxidant enzyme activity [25]. Moreover, it can disrupt various physiological functions and metabolic pathways in plants, which may, in turn, influence their stress resistance. For instance, PS-MPs can interfere with plant hormone levels and nutrient uptake, thereby compromising photosynthetic efficiency [26]. However, fewer studies have been conducted on elements related to the effects of PS-MPs on the growth of *S. lycopersicum* L. Therefore, investigating the mechanism of the effects of PS-MPs on *S. lycopersicum* L. is essential for assessing the negative impacts of microplastic contamination in soil on cash crops and developing effective pollution control strategies.

MPs significantly impact both the structure and functional dynamics of soil microbial communities. Evidence suggests that MPs influence the diversity and composition of soil microorganisms. While MPs have the potential to enhance microbial biomass, they can also lead to a reduction in microbial diversity [3]. Additionally, MPs may increase the prevalence of pathogenic microorganisms while lowering the ratio of beneficial ones. Furthermore, the impact of MPs on soil microbial communities is influenced by varying soil characteristics. For example, in acidic soils, MPs notably altered the bacterial community structure, particularly affecting the abundance of operational taxonomic units (OTUs) linked to the nitrogen cycle [27]. Under-exposure to 0.2% PS-MPs tends to impact soil fungal communities more significantly than bacterial ones [28]. MPs can also indirectly affect microbial communities by influencing soil enzyme activities. Research has shown that different categories of MPs can alter the enzymatic activities of urease, dehydrogenase, and alkaline phosphatase in the soil, which can result in notable changes to the structure of microbial communities. Specifically, there has been an observed rise in the populations of Acidobacteria and Bacteroidetes, while the abundance of Deinococcus–Thermus and Chloroflexi have diminished [29]. MPs can also alter the carbon to nitrogen ratio of the soil, thus influencing the metabolic activity of the microbial community [30]. Specifically, MPs increase the abundance of some bacteria that are able to degrade polymers, such as *TM7a*, *Phenylobacterium*, *Nocardia*, *Arthrobacter*, and *Streptomyces* [31], while the growth of some autotrophic groups like Chloroflexi and Cyanobacteria is inhibited [3]. In conclusion, the influence of MPs on soil microbial communities is multifaceted and varies greatly.

Numerous studies have examined the effects of MPs size and concentration on plant growth and soil health; however, the influence of PS-MPs on the relationships among soil health, rhizosphere microorganisms, and plants remains insufficiently investigated. This research aimed to analyze soil characteristics, rhizosphere microbial communities, and plant growth responses to clarify the impact of PS-MPs on soil ecosystems. Specifically, this study assessed how varying sizes and concentrations of PS-MPs affect *S. lycopersicum* L. and its surrounding soil. By evaluating the implications of PS-MPs on *S. lycopersicum* L. physiology, as well as soil properties and rhizosphere biology, this investigation will contribute to a deeper understanding of MPs in agricultural contexts and illuminate the related ecological risks. Additionally, the findings may provide valuable insights into reducing plastic pollution sources and promoting sustainable agricultural development and food safety.

## 2. Materials and Methods

### 2.1. Soil and MPs

Meadow soil samples were gathered from agricultural land in Cocodala City, Xinjiang Uygur Autonomous Region (81°3′15.008″ E,43°55′33.334″ N). According to the naming conventions of the IUSS Working Group WRB., 2022, the soil is classified as Histosols. After eliminating residues such as roots, leaves, and stones, the naturally air-dried soil was processed through a 200-mesh sieve. The fundamental properties of the soil were determined as follows: pH 8.82, soil organic matter (SOM) content 25.42 g·kg^−1^; ammonium nitrogen (NH_4_^+^-N) content 16.20 mg·kg^−1^; nitric nitrogen (NO_3_^−^-N) content 6.02 mg·kg^−1^; phosphorus (P) content 2.01 g·kg^−1^; available P (AP) content 58.46 mg·kg^−1^; available potassium (AK) content 18.42 mg·kg^−1^.

PS-MPs utilized in the pot experiments were sourced from Mingyuxing Plastic Material Co., Ltd. (Industrial Park Road, Dongguan, Guangdong, China), featuring particle sizes of 50 and 100 meshes. PS-MPs the PS-MPs were washed with water and dried at a temperature of 30 °C.

### 2.2. Pot Experiment

*S. lycopersicum* L. seeds were purchased from Qinshu Agriculture Co., Ltd. (Xi’an, China). For better germination, seeds underwent sterilization in a 3% hydrogen peroxide solution for 15 min, followed by rinsing with distilled water and then soaking for 24 h. Seed germination was followed by continued incubation for ten days, and then seedlings of uniform height, stem, and leaf thickness and size were transplanted into pots containing PS-MPs of vary concentrations and particle sizes. The soil used for each pot was sifted through a 200-mesh sieve. This study included five treatments: (I) CK: control treatment with no added PS-MPs; (II) PS0505: 5% (*w*/*w*) 50 mesh PS-MPs + soil; (III) PS0510: 10% (*w*/*w*) 50 mesh PS-MPs + soil; (IV) PS1005: 5% (*w*/*w*) 100 mesh PS-MPs + soil; (V) PS1010: 10% (*w*/*w*) 100 mesh PS-MPs + soil. The PS-MPs were mixed thoroughly with the soil in proportion to the total mass of 2 kg. Two plant seedlings were planted for each treatment, receiving 100 mL of water bi-daily. Each treatment was replicated thrice. Potted plants were incubated under natural light conditions for 80 days from 12 June to 31 August 2024. The average temperature during the incubation period was 25–35 °C and the light duration was 14 h. Superoxide dismutase (SOD), peroxidase (POD), and catalase (CAT) activities were determined after harvesting the plants. Rhizosphere soils were collected and preserved at −80 °C to assess soil characteristics, enzymatic activities, and microbial communities.

### 2.3. Soil Properties and Enzyme Activity Analysis

The soil analyzed for its physicochemical characteristics and enzyme activity was processed through a 200-mesh sieve. A mixture of rhizosphere soil and water (1:5, *w*:*v*) was prepared and incubated for 30 min, after which the pH was assessed using a pH meter. The soil organic carbon (SOC) was quantified using the K_2_Cr_2_O_7_ titration method, and SOM is calculated by multiplying the SOC content by the “Van Bemmelen factor” of 1.724 [32], the P concentration and AK was evaluated using the Mo-Sb colorimetric method [33]. AK, NH_4_^+^-N, NO_3_^−^-N, soil peroxidase (S-POD) activity, soil superoxide dismutase (S-SOD) activity, and soil catalase (S-CAT) activity were measured according to the instructins using the soil available potassium assay kit (BC3040), soil ammonium nitrogen content assay kit (BC1510), soil nitrate nitrogen content assay kit (BC0040), S-POD activity assay kit (BC0890), S-SOD activity assay kit (BC5130), and S-CAT activity assay kit (BC0100), respectively. All kits were obtained from Beijing Solarbio Science and Technology Co., Ltd. (Science Park Road, Beijing, Beijing, Beijing, China).

### 2.4. Plant Growth and Enzyme Activity Analysis

Harvested plants underwent a comprehensive washing process using both tap water and distilled water, followed by drying with filter paper. The roots were then carefully cut with scissors to facilitate the assessment of antioxidant enzyme activity, including SOD, POD, and CAT. These activities were evaluated using the SOD assay kit (BC0170), POD assay kit (BC0090), and CAT assay kit (BC0200) according to the manufacturer’s instructions. Plant height was recorded using a ruler. The plants were then degreened at 105 °C for 30 min and dried at 70 °C until they reached constant weight, after which their dry weights were measured.

### 2.5. Rhizosphere Microbial Community

Detailed methods for 16S rRNA sequencing of the *S. lycopersicum* L. rhizosphere soil microbial community are described in Supplementary Materials.

### 2.6. Date Analysis

Data from the experiments were subjected to analysis using one-way ANOVA followed by Tukey’s test (*p* < 0.05) with the aid of IBM SPSS Statistics 25. Significant differences among the groups are represented by distinct lowercase letters, and all data are presented as mean ± standard deviation (*n* = 6). The effects of PS-MPs on plant height, dry weight, antioxidant enzyme activity, and soil properties were evaluated. The composition of rhizosphere soil microbial communities across different treatments was analyzed using a Circos diagram, whereas a Venn diagram illustrated the relationships among the various treatments. Principal coordinate analysis (PCoA) was used to examine the variability between and within sample groups. Linear discriminant analysis effect size (LEfSe) identified biomarkers with a logarithmic LDA score of 4.0. A variance partitioning analysis (VPA) was performed to assess the impact of environmental factors on changes in community structure. Pearson correlation coefficients were calculated to explore the relationships between environmental variables and bacterial species. The Mantel test was employed to evaluate the correlation between environmental factors, genus-level bacterial species, and alpha diversity indices. Functional predictions were made using PICRUSt2. All data plotting and analyses were performed using Origin Pro 2024 and R Studio 4.4.0. A molecular ecological network analysis (MENA) was constructed based on the random matrix theory (RMT) with a threshold of 0.9000, and visualization was performed using Gephi 0.10. Partial least squares pathway model (PLS-PM) was conducted using the R package “plspm” [34].

## 3. Result and Discussion

### 3.1. Plant Growth and Antioxidant Enzyme Activity

**Figure 1:**
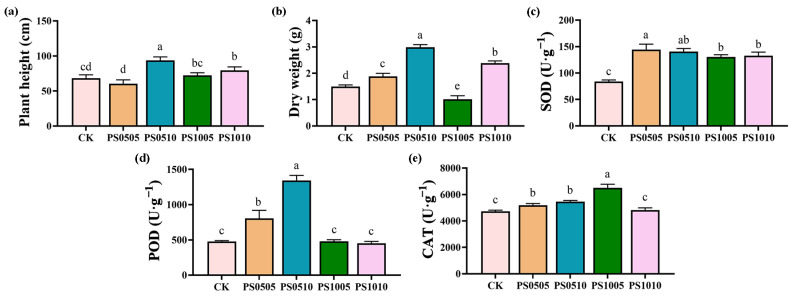
Impact of PS-MPs on growth and antioxidant enzyme activities in *S. lycopersicum* L.: (**a**) plant height; (**b**) dry weight; (**c**) SOD; (**d**) POD; (**e**) CAT. Different lowercase letters indicate significant differences, *p* < 0.05.

Plant was harvested 80 days after planting (Figure S1), after which growth and enzymatic activity were measured. Plant height and dry weight are crucial indicators that reflect the growth and development of *S. lycopersicum* L. The effects of PS-MPs on plant height and dry weight are presented in Figure 1a,b. Compared with the CK, which had a height of 66.53 cm, the PS0510 treatment resulted in a significant increase in plant height to 93.70 cm, representing a 40.83% increase. The PS1005 and PS1010 treatments also increased plant height to 72.40 cm and 79.50 cm, which represent increases of 8.82% and 19.48%, respectively. Additionally, the dry weight of seedlings was significantly enhanced in the PS0505 (1.88 g), PS0510 (2.98 g), and PS1010 (2.38 g) treatments by 25.89%, 100%, and 59.59%, respectively, compared to the CK, which had a dry weight of 1.49 g. These results suggest that different PS-MPs treatments do not exert a significant inhibitory effect on the height of *S. lycopersicum* L.; instead, they promote its growth and enhance biomass. Notably, the high-concentration with 50 mesh and 100 mesh PS-MPs treatments further increase the biomass of *S. lycopersicum* L. This finding aligns with previous research demonstrating that the addition of PS-MPs increases onion root biomass and facilitates the absorption of water and nutrients, thus promoting plant height [35]. Furthermore, the addition of 5% polyvinyl chloride MPs (PVC-MPs) has been shown to enhance wheat root exudation, aiding in nutrient and water absorption, which, in turn, supports the growth and development of wheat [36]. Similarly, various types and concentrations of MPs have been reported to increase the biomass of both carrot plant shoots and roots [37].

The activities of plant roots antioxidant enzymes, including SOD, POD, CAT, are presented in Figure 1c–e. Compared to the CK, the incorporation of PS-MPs had varied effects on the activities of these antioxidant enzymes in plants. Specifically, SOD activity significantly increased in the PS0505 (144.44 U·g^−1^), PS0510 (140.59 U·g^−1^), PS1005 (130.56 U·g^−1^), and PS1010 (132.88 U·g^−1^) treatments, with increases of 72.26%, 67.66%, 55.71%, and 58.48%, respectively, compared to CK (83.84 U·g^−1^). In addition, the PS0505 (804.34 U·g^−1^) and PS0510 (1343.60 U·g^−1^) treatments significantly enhanced POD activity by 68.82% and 182.01%, respectively, compared to the CK (476.42 U·g^−1^). PS0510 (5454.3 U·g^−1^) and PS1005 (6497.2 U·g^−1^) showed considerable increases in CAT (catalase) activity by 15.42% and 37.48%, respectively, compared to CK (4725.6 U·g^−1^). The activity of plant antioxidant enzymes can mitigate oxidative stress damage caused by the production of reactive oxygen species (ROS) and free radicals [38]. The addition of PS-MPs significantly enhanced the antioxidant enzyme activity in *S. lycopersicum* L. By boosting antioxidant enzyme activity, *S. lycopersicum* L. protects its cells from oxidative stress. This increase in enzyme activity not only safeguards *S. lycopersicum* L. growth but may also contribute to the observed increase in *S. lycopersicum* L. biomass. Furthermore, MPs have been found to induce oxidative stress in cotton roots, leading to enhanced antioxidant enzyme activity [39]. Research indicates that when the mass fraction of polyethylene MPs reaches 7% and 14%, the activity of CAT is significantly elevated compared to the CK [40]. Enhanced antioxidant enzyme activity may also support plant growth and development under stress conditions [41].

### 3.2. Soil Physicochemical/Biochemical Properties

**Figure 2:**
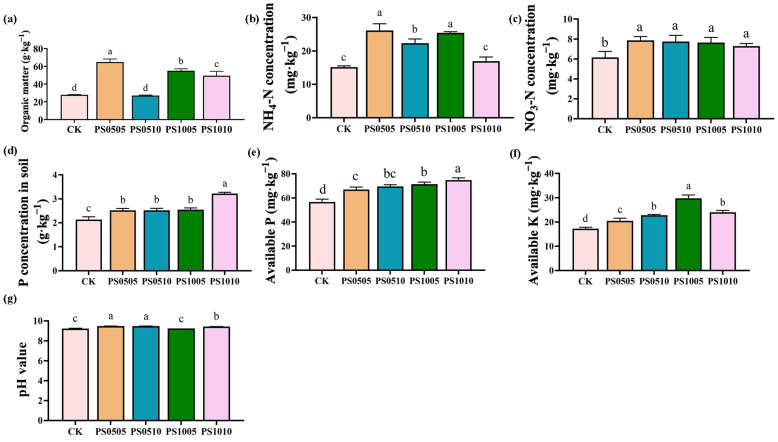
Physicochemical of soil: (**a**) organic matter; (**b**) NH_4_-N concentration; (**c**) NO_3_-N concentration; (**d**) P concentration; (**e**) available P; (**f**) available K; (**g**) pH value. Different lowercase letters indicate significant differences, *p* < 0.05.

Soil physicochemical properties, including SOM, NH_4_-N, NO_3_-N, and P concentrations, AK, AP, and pH, are depicted in Figure 2a–e. The results indicate that compared with the CK (27.81 g·kg^−1^), treatments with PS0505 (64.94 g·kg^−1^), PS1005 (55.01 g·kg^−1^), and PS1010 (49.51 g·kg^−1^) significantly increased SOM by 133.46%, 97.76%, and 77.99%, respectively (Figure 2a). SOM is a critical indicator of soil fertility and plays a vital role in maintaining ecological stability. This increase in SOM may be attributed to the higher carbon content in PS-MPs, which introduces exogenous carbon sources into the soil, thereby enhancing organic carbon content. Previous studies have demonstrated that polyethylene MPs (PE-MPs) at a mass fraction of 1% can elevate SOM content by 72% to 324% [42]. These findings align with earlier research, which further suggests that MPs can indeed enhance SOM content.

N, P, and K are essential nutrients in the soil, significantly influencing the growth and metabolism of soil microorganisms. The NH_4_-N content compared to CK (15.15 mg·kg^−1^) increased in the PS0505 (26.15 mg·kg^−1^), PS0510 (22.33 mg·kg^−1^), and PS1005 (25.44 mg·kg^−1^) treatments by 72.66%, 47.42%, and 67.95%, respectively (Figure 2b). The NO_3_^−^-N content increased in the treatments compared to the control group (CK, 6.16 mg·kg^−1^). Specifically, the PS0505 treatment showed an increase to 7.86 mg·kg^−1^ (27.60%), PS0510 reached 7.75 mg·kg^−1^ (25.81%), PS1005 increased to 7.65 mg·kg^−1^ (24.19%), and PS1010 reached 7.29 mg·kg^−1^ (18.34%) (Figure 2c). This enhancement may result from MPs influencing the activity of enzymes associated with the soil nitrogen cycle and altering the proportions of nitrogen-fixing microorganisms, thus affecting soil nitrogen content [43]. For instance, a 0.5% polylactic acid (PLA) treatment has been shown to increase soil enzyme activity and NH4^+^ concentration, leading to a boost in nitrogen content [44]. Additionally, MPs can also affect soil denitrification through changes to the pore structure and aggregate morphology of the soil [14]. They may promote the growth and reproduction of nitrogen-fixing bacteria, which further contributes to the increased soil nitrogen content [45]. The treatments PS0505 (2.5185 g·kg^−1^), PS0510 (2.5174 g·kg^−1^), PS1005 (2.5466 g·kg^−1^), and PS1010 (3.2184 g·kg^−1^) resulted in significant increases in soil P concentrations of 18.09%, 18.04%, 19.41%, and 50.91%, respectively, compared with CK (2.1326 g·kg^−1^) (Figure 2d). Similarly, the AP content also increased in these treatments compared to CK (56.61 mg·kg^−1^). The PS0505 treatment recorded a content of 66.90 mg·kg^−1^ (18.17%), PS0510 reached 69.51 mg·kg^−1^ (22.79%), PS1005 increased to 71.34 mg·kg^−1^ (26.02%), and PS1010 showed a final value of 74.72 mg·kg^−1^ (31.99%) (Figure 2e). Studies have shown that high concentrations of MPs stimulate the activities of extracellular enzymes related to phosphorus cycling [46]. Furthermore, MPs may alter soil P content through adsorption processes. Similarly, the AK content compared to CK (17.21 mg·kg^−1^) significantly increased in the PS0505 (20.48 mg·kg^−1^, 19.01%), PS0510 (22.78 mg·kg^−1^, 32.39%), PS1005 (29.76 mg·kg^−1^, 72.96%), and PS1010 (24.00 mg·kg^−1^, 39.47%) treatments (Figure 2f). This effect is likely due to MPs interfering with potassium release by modifying soil structure, as MPs can alter soil aggregate structure and thus affect potassium release capacities, ultimately increasing the available potassium content in rice soil [47].

Finally, the pH of the soil was altered by the treatments, with PS0505 (9.48), PS0510 (9.47), PS1005 (9.24), and PS1010 (9.42) showing increases from CK (9.23) of 2.68%, 2.63%, 0.12%, and 2.11%, respectively (Figure 2g). The increase in soil pH treated with PS-MPs may be related to the decomposition of MPs, caused by the hydrolysis of carbonate ions released into the soil solution, leading to an increase in pH [48]. Additionally, changes in soil pH may be associated with modifications in the soil biological community. LDPE-MPs have been found to increase soil pH by altering the nitrification processes and the abundance of ammonia-nitrifying bacteria, which release H^+^ ions [49]. However, it is notable that MPs may also cause a decrease in soil pH, potentially related to cation exchange in the soil and alterations in the state of free proton exchange in soil water [50].

**Figure 3:**
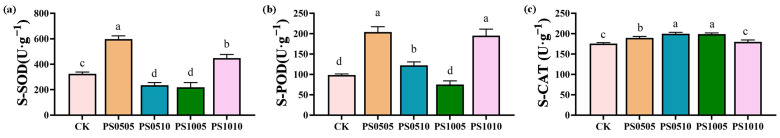
Biochemical of soil (**a**) S-SOD; (**b**) S-POD; (**c**) S-CAT. Different lowercase letters indicate significant differences, *p* < 0.05.

Soil antioxidant enzyme activities, including S-SOD, S-POD, S-CAT, are shown in Figure 3a–c. The incorporation of polystyrene MPs (PS-MPs) had notable effects on soil antioxidant enzyme activities when compared to the CK. Specifically, the PS0505 treatment (597.11 U·g^−1^) and PS1010 treatment (447.92 U·g^−1^) resulted in significant increases in S-SOD activity, with increases of 83.84% and 37.90%, respectively, relative to CK (324.79 U·g^−1^) (Figure 3a). Furthermore, S-POD activity was significantly enhanced by the PS0505 (204.24 U·g^−1^), PS0510 (122.30 U·g^−1^), and PS1010 (195.14 U·g^−1^) treatments, which resulted in increases of 107.4%, 24.19%, and 98.16%, respectively, compared to CK (98.47 U·g^−1^) (Figure 3b). In terms of S-CAT activity, the application of PS0505 (189.5 U·g^−1^), PS0510 (199.7 U·g^−1^), PS1005 (199.0 U·g^−1^), and PS1010 (179.9 U·g^−1^) resulted in increases of 7.78%, 13.61%, 13.22%, and 2.33%, respectively, compared to CK (175.8 U·g^−1^) (Figure 3c).

Soil antioxidant enzymes play a crucial role in effectively removing reactive oxygen species (ROS) to mitigate oxidative damage, regulating soil microorganisms, and maintaining ecological balance within the soil. As indicated by the results (Figure 3a–c), the incorporation of PS-MPs with different concentration and size led to significant change in soil antioxidant enzyme activity, highlighting their potential role in enhancing soil health.

### 3.3. Diversity and Composition of Rhizosphere Soil Microbial Communities

**Figure 4:**
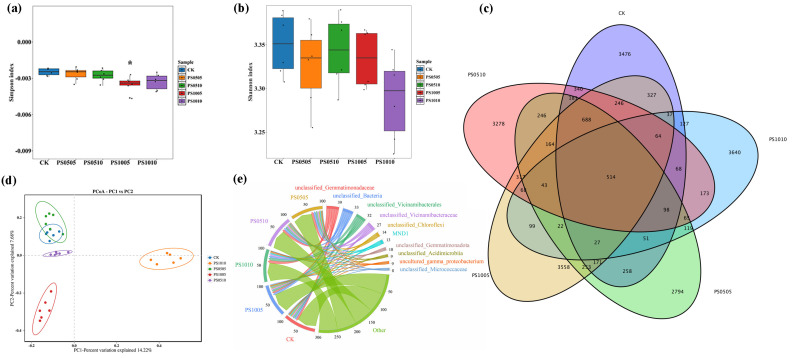
Diversity and composition of rhizosphere soil bacterial community in *S. lycopersicum* L: (**a**) Simpson Index; (**b**) Shannon Index; (**c**) Venn diagram; (**d**) PCoA; (**e**) the abundance of microorganisms at the genus level in a Circos diagram. “*” represent significant differences (*p* < 0.05).

The Simpson and Shannon index (Figure 4a,b) were employed to evaluate the diversity of rhizosphere soil microbial communities across five different treatments. The results indicated a significant enhancement in microbial diversity in the PS1005 treatment relative to the CK, whereas no significant variations were detected in the other treatments. Although a higher Shannon index typically signifies greater microbial diversity, no significant effects were identified in the present results. The Venn diagram shows that the number of OTUs between CK, PS0505, PS0510, PS1005, and PS1010 is 534, while the distinct OTUs for each treatment are 5476, 2794, 3278, 3558, and 3640, respectively (Figure 4c). This result indicates that PS-MPs with larger particle sizes have a more significant impact on the OTU numbers. However, the negative effect of higher concentrations on OTU numbers is smaller negative at the same particle size. PCoA was performed using Bray–Curtis distances to assess the rhizosphere soil microbial communities across the different treatments (Figure 4d). The analysis revealed that the principal components (Pc1 and Pc2) contributed 14.22% and 7.68% to the overall microbial diversity, respectively, resulting in a cumulative contribution of 21.9%. This analysis demonstrated notable differences among the groups, while the variations observed within each group were comparatively minimal.

The sequencing of 16S rRNA revealed a total of 40 phyla, 102 orders, 296 families, 575 genera, and 21,513 OTUs. Variations in particle size and concentration of PS-MPs led to changes in bacterial community composition across different taxonomic levels. At the genus level (Figure 4e), the major bacteria included *unclassified_Gemmatimonadaceae* (5.95–11.93%), *unclassified_Bacteria* (5.35–7.57%), *unclassified_Vicinamibacterales* (4.90–8.77%), *unclassified_Vicinamibacteraceae* (4.34–6.83%), *unclassified_Chloroflexi* (1.58–3.57%), MND1 (1.79–4.10%), *unclassified_Gemmatimonadota* (1.42–2.80%), *uncultured_gamma_proteobacterium* (1.27–2.95%), and *unclassified_Micrococcaceae* (1.06–2.35%). Compared to the CK, the application of PS-MPs resulted in a rise in the relative abundance of *unclassified_Acidimicrobiia* and decreasing *unclassified_Chloroflexi* and *MND1* (Table S1). In addition, although the response of some microbial communities was not significant compared to CK, significant changes were found between experimental groups. For example, the abundance of *unclassified_Gemmatimonadaceae*, *unclassified_Vicinamibacterales*, and *unclassified_Vicinamibacteraceae* was higher was higher in the PS1010 compared to the PS1005. At the phylum level (Figure S2), the bacterial community composition was predominantly comprised of Proteobacteria (16.79–24.33%), Acidobacteriota (15.91–25.26%), Actinobacteriota (10.51–20.79%), Gemmatimonadota (8.82–17.62%), Chloroflexi (5.86–9.05%), unclassified_Bacteria (5.35–7.57%), Myxococcota (3.69–5.91%), Bacteroidota (2.77–6.97%), and Patescibacteria (1.57–3.85%), among others. In comparison to the CK, the presence of PS-MPs led to a decrease in the relative abundance of *Proteobacteria* and *Myxococcota*, and the relative abundance of *Gemmatimonadota* and *Patescibacteria* increased (Table S2). At the family level, he prominent bacterial communities included *Gemmatimonadaceae* (6.50–13.75%), *unclassified_Vicinamibacterales* (5.88–10.73%), *Vicinamibacteraceae* (5.10–7.54%), *Nitrosomonadaceae* (2.43–5.87%), *unclassified_Chloroflexi* (1.58–3.57%), *Sphingomonadaceae* (1.69–2.58%), and *Micrococcaceae* (1.13–2.71%) et al. (Table S3).

Microorganisms are essential contributors to the biogeochemical cycling of nutrients within the soil. The findings indicate that PS-MPs are capable of altering both the diversity and structural composition of microbial communities within the soil. Multiple investigations have emphasized the beneficial impacts of PS-MPs on microbial populations, primarily due to their role as carriers offering attachment points for microorganisms, thus promoting biofilm formation [50]. Furthermore, PS-MPs may function as a potential source of carbon, which could enhance the growth of particular microbial communities [51]. Other investigations have identified *unclassified_Gemmatimonadaceae*, *unclassified_Vicinamibacterales*, and *unclassified_Vicinamibacteraceae* as dominant microorganisms in the soil [52,53]. The metabolic activity of unclassified *Gemmatimonadaceae* is known to enhance soil nutrient content, which can positively influence plant growth and soil functionality [54]. Furthermore, unclassified_*Vicinamibacteraceae* has been shown to participate in the nitrogen cycle, playing a significant role in improving soil fertility [55]. Conversely, oxidative stress induced by PS-MPs can cause oxidative damage to certain microorganisms, resulting in a decline in their abundance [56]. PS-MPs also affect microbial metabolism, leading to alterations in community abundance and diversity [57]. Conversely, oxidative stress induced by PS-MPs can cause oxidative damage to certain microorganisms, resulting in a decline in their abundance. PS-MPs also affect microbial metabolism, leading to alterations in community abundance and diversity. 

**Figure 5:**
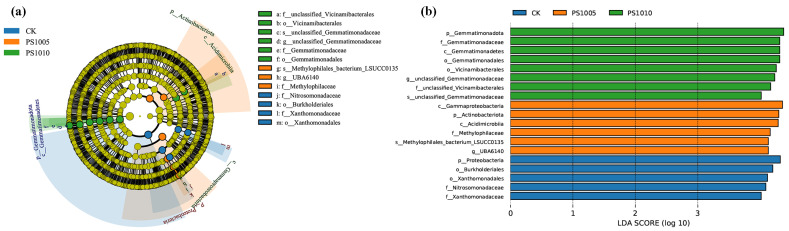
Linear discriminant analysis effect size (LEfSe): (**a**) diagram depicting the evolutionary branching; (**b**) histogram displaying the distribution.

To evaluate the impact of different concentrations and particle sizes of PS-MPs on the soil microbial community, LEfSe analysis was conducted, examining the community from the phylum to species levels (Figure 5). A total of 13 biomarkers were identified across seven taxonomic levels in both the CK and PS-MP treated samples (Figure 5a). Specifically, biomarkers were found at the following levels: 2 at the species level; 2 at the genus level; 5 at the family level; 4 at the order level; 3 at the class level; and 3 at the phylum level (Figure 5b). Significant differences were observed in the microbial communities of soils treated with varying PS-MP concentrations compared to the CK, with six biomarkers showing notable increases. Biomarkers 6 and 8 were uniquely associated with the PS1005 and PS1010 treatments, respectively. In the CK group, species from the Proteobacteria phylum were relatively abundant, while Gammaproteobacteria and Actinobacteriota were more prevalent in the PS1005 treatment, and Gemmatimonadota was more abundant in the PS1010 treatment. Biomarkers serve as important indicators of soil microbial community structure and function and can be used to assess the abundance and composition of major soil microbial communities [58]. Proteobacteria are known for their rich diversity of microbial species due to their critical role in soil functionality; they are the most abundant phylum involved in the biogeochemical cycling of important soil nutrients like N, P and sulfur [59]. The enrichment of Actinobacteriota in the PS1005 treatment may be attributed to selective enrichment by microorganisms. Some studies have demonstrated that actinomycetes can replace Proteobacteria as the dominant phylum in exposed soils after the addition of 5% polyethylene MPs, indicating that MPs exert selective pressure on microorganisms and alter the diversity and richness of microbial communities. Additionally, Gemmatimonadota exhibits a strong adaptive capacity in the presence of PS-MPs.

### 3.4. Co-Occurrence Networks Analysis

**Figure 6:**
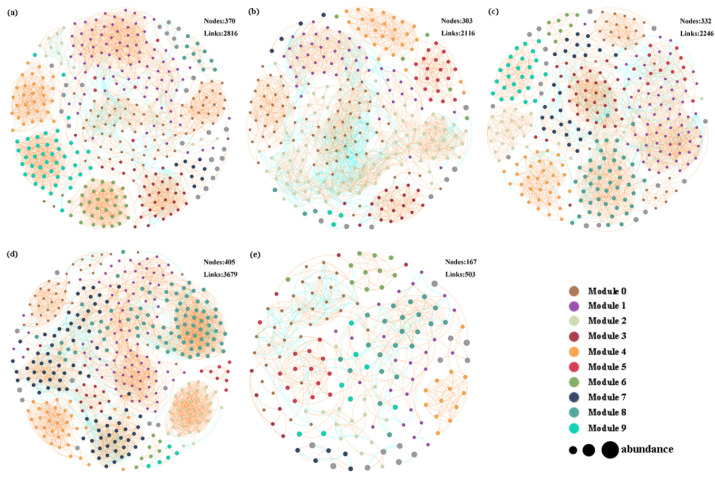
Co-occurrence Networks analysis: (**a**) CK; (**b**) PS0505; (**c**) PS0510; (**d**) PS1005; (**e**) PS1010.

The molecular ecological network derived from 16S rRNA sequencing data across five treatment groups was analyzed using the random matrix theory (RMT) network method, resulting in the construction of five distinct networks (Figure 6a–e). The primary topological properties of the microbial community molecular ecological network analysis (MENA) were summarized in Table S4. With a consistent threshold of 0.900, the power law R-squared values for the various treatments were as follows: CK at 0.219, PS0505 at 0.188, PS0510 at 0.219, PS1005 at 0.186, and PS1010 at 0.437. The number of nodes for each treatment group was as follows: CK (370), PS0505 (303), PS0510 (332), PS1005 (405), and PS1010 (167). In this case, in comparison, CK exhibited a greater number of links (2816), a higher percentage of positive links (94.39%), and a lower percentage of negative links (5.61%) than the other treatments: PS0505 (2116 links, 81.66% positive, 18.34% negative), PS0510 (2246 links, 90.74% positive, 9.26% negative), and PS1010 (503 links, 76.54% positive, 23.46% negative). These results indicate that the introduction of MPs significantly disrupts the interactions within bacterial communities, leading to a substantial decline in microbial diversity, particularly in the PS1010. PS-MPs with small particle size and high concentration and large particle size and low concentration had more serious effects on microbial community structure. Conversely, the PS1005 treatment demonstrated the highest interaction level, with a total of 3679 links (92.12% positive and 7.88% negative), suggesting that smaller particle size and lower concentrations of PS-MPs enhance microbial interactions and increase network complexity. The ZP analysis identified key operational taxonomic units (OTUs) as follows: one key OTU in CK, one in PS0505, three in PS0510, and one in PS1005 (Figure S5). These key nodes predominantly belonged to the phyla *Proteobacteria*, *Bdellovibrionota*, *Actinobacteriota*, and *Acidobacteriota*.

This study established five co-occurrence networks to examine the influence of PS-MPs with varying concentrations and particle sizes on the interrelations among microbial communities. In comparison to the CK, the introduction of PS-MPs resulted in a decrease in both positive correlation links and overall connections among microbial communities. This decrease indicates a reduction in the complexity of microbial communities in response to PS-MPs, as well as markedly differing enrichment and inhibition effects across various microbial populations [29,60]. The reduction in links among microbial communities signifies diminished correlations, suggesting that microbial populations are behaving more competitively [61]. Furthermore, a significant reduction in positive correlation links alters the structure and functioning of microbial communities, thereby compromising their stability [62]. Consequently, this disruption renders the ecosystem increasingly vulnerable. The underlying cause of these phenomena is the lack of key microbial nodes. Prolonged exposure of certain bacteria within the Proteobacteria phylum to soils amended with PS-MPs negatively affects their metabolic pathways. This includes downregulation of critical processes such as photosynthesis, carbon metabolism, amino acid metabolism, lipid synthesis, and nucleoside metabolism [63]. The interactions of *Bdellovibrio* with other bacteria are not only limited to predatory relationships, and their abundance was similar to the trend of abundance changes of other bacterial phyla in the ecosystem, such as Peredibacteraceae. This suggests that *Bdellovibrio* may influence community structure through its interactions with other microorganisms within the ecosystem [64]. In the plant rhizosphere, Actinobacteria collaborate closely with host plants and other bacterial groups, such as Firmicutes and Acidobacteria, to provide beneficial functions that enhance plant disease resistance [65]. Moreover, Acidobacteriota play a significant role in organic carbon degradation and actively contribute to the soil ecosystem’s health and functionality [66].

### 3.5. Correlation Analysis

**Figure 7:**
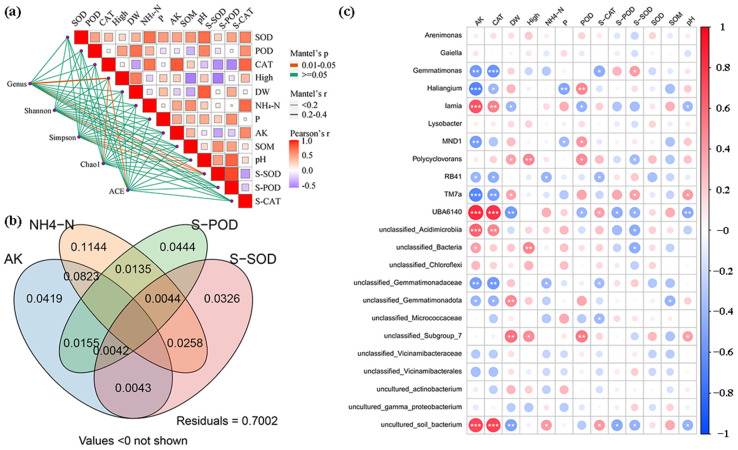
Correlation analysis of bacterial communities, plant growth, enzyme activities and the physicochemical and biochemical properties of soil: (**a**) Mantel’s test investigated the correlation between the genus level bacteria and the indices of Shannon, Simpson, Chao1, and ACE; (**b**) VPA analysis; (**c**) Pearson correlations of genus, soil physicochemical, and biochemical properties. “*”, “**” and “***” represent significant differences (*p* < 0.05, *p* < 0.01 and *p* < 0.001).

The results of the Mantel test analysis (Figure 7a) revealed a positive correlation between the Shannon and Simpson diversity index and S-SOD activity. Moreover, a positive relationship was detected between both the Chao1 and ACE indices with high and S-SOD activity. A significant positive correlation also existed among the antioxidant enzymes (SOD, POD, and CAT) and both NH_4_-N and S-CAT; conversely, there was a noteworthy negative correlation between CAT with S-SOD and S-POD. A significant positive relationship was observed between High and DW, while a significant negative correlation emerged between both and SOM. There was a significant positive correlation between DW and pH, a significant positive correlation between NH_4_-N and AK, SOM, and S-CAT, a significant positive correlation between P and AK and S-POD, a significant positive correlation between AK and S-CAT, a significant negative correlation with S-SOD, a significant positive correlation between S-SOD and SOM and pH, and a significant negative correlation with S-CAT. There was a significant positive correlation between pH and S-SOD. The VPA depicted in Figure 7b indicate that the combined effect of the four variables, AK, NH_4_-N, S-POD, and S-SOD, accounts for an effect value of 0.0043. The individual effect values for these variables are as follows: AK at 0.1144, NH_4_-N at 0.0419, S-POD at 0.0444, and S-SOD at 0.0326. The residual value is notably high, at 0.7002, suggesting that the combined influence of AK, NH_4_-N, S-POD, and S-SOD accounts for only a minimal portion of the overall variation, with a substantial 70.02% remaining unexplained. These data show that NH_4_-N, as a nitrogen source, can stimulate the growth of some microbial communities. Under the action of microorganisms, the conversion process of NH_4_-N to NO_2_-N is accelerated, which helps to increase the abundance of the microbial community and thereby Form a virtuous circle [67]. At the same time, NH4-N plays an important role in explaining variations in soil chemical properties [68], which is related to its key role in soil fertility and plant nutrition [69]. AK is essential for regulating cellular energy metabolism, particularly in bacteria. By catalyzing the reversible phosphorylation transfer reaction between ATP and AMP, AK maintains the ratio of adenosine nucleotides within the cell, which is vital for bacterial growth, differentiation, and movement [70]. The oxygen generated by S-POD through its catalytic activity can promote the activity of aerobic microorganisms, thereby accelerating microbial activities and process performance [71]. S-SOD converts superoxide radicals into oxygen and hydrogen peroxide, which is critical for microorganisms facing oxidative stress [72]. It also protects microorganisms from oxidative damage and influences their metabolic pathways and physiological status [73]. Furthermore, S-SOD enhances the organic matter and total nitrogen content in the soil by improving its physical and chemical properties, thereby increasing the soil’s nutrient retention capacity and providing a more favorable growth environment for soil organisms [74].

The correlation heatmap (Figure 7c) illustrates the relationships between environmental factors and various genera. *Gemmatimonas*, *unclassified_Gemmatimonadaceae*, and *unclassified Gemmatimonadota* exhibited significant negative correlations with AK and CAT. Additionally, a negative correlation was observed between *unclassified_Gemmatimonadaceae* and *RB41* concerning NH_4_-N. In contrast, Desulfobacterota, Patescibacteria, Spirochaetota, and Thermoplasmatota displayed significant positive correlations with both AK and CAT, whereas Armatimonadota and Gemmatimonadota showed significant negative correlations with these variables. Abditibacteriota was positively correlated with DW, High, and POD, while Dependentiae exhibited a significant negative correlation with POD. The positive correlations of Desulfobacterota, Patescibacteria, Spirochaetota, and Thermoplasmatota with AK and CAT may be linked to the effects of microplastic treatment on microbial activity and, consequently, on AK content (Figure S4). This suggests that soil microorganisms may experience stress due to MPs, leading to reduced enzyme synthesis and lower nitrogen levels in the soil. Previous studies have shown that polystyrene nanoparticles can inhibit microbial abundance and soil enzyme activities, thereby disrupting the metabolic functions of soil microorganisms and affecting the nitrogen cycle [75]. AK and CAT displayed significant positive correlations with genera such as *Lamia*, *UBA6140*, *unclassified_ Acidimicrobiia*, and *uncultured*_*soils bacterium*, while demonstrating significant negative correlations with *Haliangium*, *RB41*, and *TM7a*. Furthermore, both DW and High had significant positive correlations with *Polycyclovorans* and unclassified *Subgroup_7*. Soil microorganisms capable of solubilizing potassium can release soluble potassium from minerals and rocks, thereby increasing its availability in the soil [76]. Additionally, these microorganisms enhance plant growth and development by improving the rhizosphere microenvironment. Research has indicated that the addition of 7.5% MPs and 2% bio compost increases the abundance of bacteria and fungi in the rhizosphere, as well as the nitrogen, phosphorus, and carbon content in the soil, facilitating nutrient absorption and promoting plant growth [77]. Structural changes in microbial communities may be closely linked to antioxidant response mechanisms, influencing their ability to adapt to environmental stressors. MPs can induce oxidative stress by elevating levels of reactive oxygen species (ROS) and altering antioxidant enzyme activity, prompting microorganisms to deploy various antioxidant defense mechanisms in response [78]. Notably, there is a significant negative correlation between soil pH and *UBA6140*, suggesting that changes in bacterial communities may be associated with alterations in soil pH [79]. Additionally, a negative correlation exists between soil pH and the relative abundance of certain microorganisms. It is plausible that chemicals released through the biochemical processes of MPs in the soil stimulate microbial activity, resulting in changes to soil pH [80].

### 3.6. Effects of PS-MPs on the Soil–Microbial–Plant System

**Figure 8:**
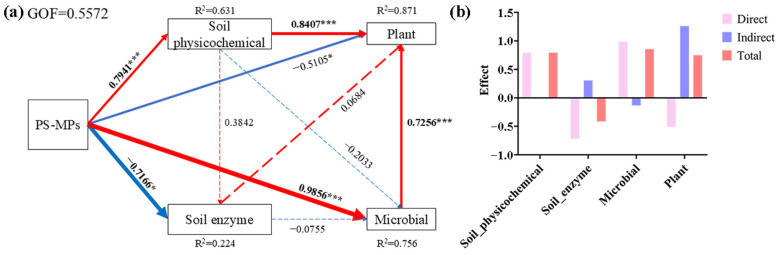
PLS-PM showing the direct and indirect effects of adding PS-MPs to the soil–microbe–plant system. The width of arrows is proportional to the strength of the pathway coefficients. Red and blue arrows indicate positive and negative causality, respectively, with solid lines representing significant effects (*p* < 0.05), and dashed lines representing non-significant effects (*p* > 0.05). “*” indicates *p* < 0.05; “***” indicates *p* < 0.001. (**a**) PLS-PM; (**b**) Effect of direct, indirect and total. Soil physicochemical effects included NH_4_-N, P, AK, SOM, and pH; soil enzymes included S-SOD, S-POD, and S-CAT; microbial effects included the Simpson Index and Shannon Index; plant effects included SOD, POD, CAT, Height, and Dry weight.

The PLS-PM analysis results highlight the complex relationships among soil physicochemical properties, soil enzyme activities, microbial communities, and plant growth (Figure 8). The model’s overall goodness-of-fit (GOF) is moderate at 0.5572. The model explains 22.4% of the variance in soil physicochemical properties and 38.4% of soil enzyme activities, while it has a strong explanatory power for microbial communities (76.5%) and plant (72.56%). PS-MPs have a significant positive effect on soil physicochemical properties (0.7941) and soil microbial community diversity (0.9856). The results of [81] suggest that the addition of MPs can improve soil quality and microbial diversity and may be able to promote plant growth. And there is a significant negative direct effect on soil enzyme activities (−0.7166) and plant growth (−0.5105). The high concentrations of PS-MPs may also have detrimental effects, possibly by disrupting enzyme functions and directly inhibiting plant growth. Despite the negative direct effects, the total effect of PS-MPs on plant growth is positive (Figure 8b). The contrasting total effect on plants implies that other indirect mechanisms may be at play, mitigating the negative direct effects. The strong positive direct effects are also evident from soil physicochemical properties to plant growth (0.8407) and from microbial communities to plant growth (0.725). These findings underscore the importance of soil physicochemical properties and microbial communities in influencing plant growth, with PS-MPs playing a dual role in both positive and negative influences [82,83].

### 3.7. Examination of Predictive Functional Genes

**Figure 9:**
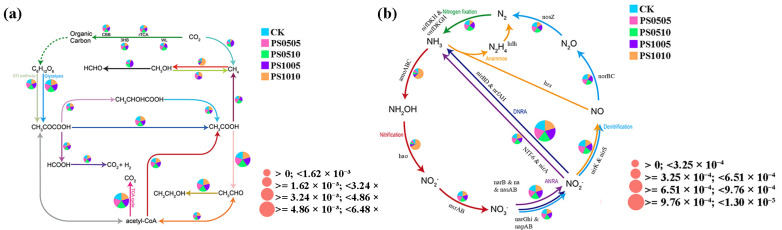
Relative abundance of the pathways involved in the C and N cycle. (**a**) C cycle; (**b**) N cycle. The pie chart illustrates the proportional representation of various pathways within each metagenomic sample. The dimensions of the pie charts reflect the overall relative abundance of each pathway.

Gene function prediction was conducted using 16S rRNA sequencing data to investigate the impact of PS-MPs on C and N cycling pathways in microbial communities. The results of the carbon cycle function prediction (Figure 9a) indicated that varying contents and particle sizes of PS-MPs influence different reaction processes in the carbon cycle. Notably, the effects of various treatments on genes and pathways related to methane production and transformation were significant (Table S5). Specifically, PS1005 elevated the expression of mcrABG, enhancing the microbial conversion of CO_2_ to methane, while treatments PS0505 and PS0510 produced contrary results. PS0510 increased cdhCDE expression and promoted microbial conversion of acetic acid to methane, while PS1005 enhanced the conversion of carbon dioxide to methane. Treatment with PS1010 elevated the expression of mmoBCDXYZ and amoABC, facilitating the methanolization process. Furthermore, PS1005 increased the expression of mxaFI and xoxF, promoting the conversion of methanol to formaldehyde. Previous studies have also highlighted that cdhCDE overexpression improves the ability of acetyl-CoA to accelerate methyl reduction to methane [84,85]. The upregulation of these genes enhances methanogen functionality, thereby accelerating acetic acid degradation and facilitating methane production [86]. Enhanced expression of the mtaABC gene promotes methyltransferase activity and augments the production of the methyltransferase complex, which accelerates methanol metabolism and facilitates its conversion to methane [87]. Both mmoBCDXYZ and amoABC can influence the oxidation of methane to methanol [88].

Under PS1010 treatment, a notable reduction was observed in the relative abundance of denitrification pathway genes, including *nirK*, *nirS, norBC*, and *nosZ* (Table S6). In contrast, PS1005 treatment led to an increased relative abundance of genes associated with the assimilative nitrate reduction pathway, such as *narB*, *nasAB*, *NIT-6*, and *nirA*. The PS0510 treatment decreased the abundance of dissimilatory nitrate reduction pathway genes (*NarGHI*, *napAB*, *nirBD*, *nrfAH*), while the PS1010 treatment heightened the relative abundance of nitrification pathway genes (*amoABC*), although the abundance of hao and *nxrAB* pathway genes decreased. Furthermore, PS1010 treatment resulted in a decline in the relative abundance of nitrogen fixation pathway genes (*nifDKH*). Compared to the CK, microplastic treatment significantly increased the overall abundance of assimilative nitrate reduction genes. These findings indicate that PS-MPs substantially diminish the expression of functional genes related to denitrification, likely due to alterations in microbial community structure and function. Previous studies have shown decreases of 70.7% and 56.3% in the abundance of *nirK* and *nirS* genes, respectively, in rice soil under polyethylene MPs stress [47]. However, other research has indicated that polyethylene MPs treatment can also increase the abundance of *nirK* and *nirS* genes [89]. This discrepancy may be attributed to differences in soil types. Microorganisms play a crucial role in reducing nitrate to nitrite, participating in the assimilative nitrate reduction process [90]. Some studies have demonstrated that the addition of polyurethane foam and polylactic acid can enhance nitrate and nitrite concentrations in sediment, subsequently increasing the nitrification rate [91]. The influence of PS-MPs on the abundance of nitrification-related genes may depend on microplastic particle size and soil porosity; increased porosity can facilitate the movement of oxygen molecules, thereby enhancing nitrification [92]. The PS1010 treatment’s reduction in nitrogen-fixing gene abundance may also be related to the varying doses of MPs administered. Research indicates that nitrogen fixation and denitrification genes in the soil experience a more pronounced decrease at an 8% PE-MP addition rate compared to a 1% addition rate [93]. Conversely, high phthalate content in polyvinyl chloride MPs can significantly increase the abundance of *nifDKH* genes, thereby enhancing nitrogen fixation pathway gene expression [43]. This finding diverges from ours and may be attributed to the different types of MPs studied. In summary, MPs can influence the expression of microbial functional genes involved in C and N cycles, resulting in various changes in gene forms.

## 4. Conclusions

The present study elucidates the multifaceted effects of PS-MPs on the growth and physiological responses of *S. lycopersicum* L., as well as their influence on soil physicochemical properties and microbial communities. Our findings demonstrate that PS-MPs significantly stimulate plant growth, with the most pronounced effects observed in treatments with higher concentrations of PS-MPs. This growth promotion is accompanied by an enhancement in antioxidant enzyme activities, indicating that PS-MPs may induce plant stress responses that contribute to growth enhancement. Soil physicochemical analyses reveal that PS-MPs increase soil organic matter and nutrient content, which are crucial for soil fertility and plant nutrition. The microbial community structure and diversity are also significantly altered by PS-MPs, with shifts in bacterial composition and functional gene expression related to carbon and nitrogen cycling. These changes underscore the complex interactions between PS-MPs, soil properties, and microbial communities, the importance of soil physicochemical properties and microbial communities in influencing plant growth was also emphasized through PLS-PM, in which PS-MPs play a dual role in positive and negative effects. In conclusion, our study provides insights into the role of PS-MPs in modulating the soil–microbial–plant system, highlighting their potential to alter soil fertility, microbial community structure, and plant physiological responses. These findings contribute to the understanding of the ecological implications of microplastic pollution and suggest avenues for further research into the development of mitigation strategies for soil and plant health. Importantly, we recommend that future research should focus on the specific effects of MPs within the rhizosphere soil of *S. lycopersicum* L. This should include experimental designs that explore various concentrations and types of MPs, as well as their potential effects on root exudates and microbial interactions. Such studies will enhance our understanding of their implications for microbial ecosystems and provide a more comprehensive assessment of the impacts of microplastic pollution in agricultural contexts.

## Data Availability

16S rRNA sequencing data were deposited in the Sequence Read Archive (SRA) of the National Center for Biotechnology Information (NCBI) under accession number PRJNA1190609.

## Supplementary Materials

The following supporting information can be downloaded at https://www.mdpi.com/article/10.3390/plants14020256/s1, Figure S1: The harvested *Solanum lycopersicum* L. after 80 days; Figure S2: The abundance of microorganisms at the phylum level; Figure S3: The abundance of microorganisms at the family level; Figure S4: Spearman correlations of phyla, soil and *Solanum lycopersicum* L. parameters; Figure S5: ZP diagram; Table S1: The relative abundances at genus in the five groups; Table S2: The relative abundances at phylum in the five groups; Table S3: The relative abundances at family in the five groups; Table S4: Major topological properties of empirical MENs microbial communities in the five groups; Table S5: The relative abundance of key functional genes about N cycle in different microbial communities; Table S6: The relative abundance of key functional genes about N cycle in different microbial communities.
