# Supplementary material for: Impact of Polystyrene Microplastics on Soil Properties, Microbial Diversity and Solanum lycopersicum L. Growth in Meadow Soils
Source: Plants (Basel). 2025 Jan 17;14(2):256. doi: 10.3390/plants14020256 (PMC11768701; doi:10.3390/plants14020256)
Supplement: Supplementary file 1 [file plants-14-00256-s001.zip › plants-3420686-supplementary.pdf]

Impact of Polystyrene Microplastics on Soil Properties, Microbial Diversity and *Solanum lycopersicum* L. Growth in Meadow Soils

Materials and methods

Rhizosphere microbial community

DNA was performed utilizing the TGuide S96 Magnetic Soil/Stool DNA Kit from Tiangen Biotech (Beijing) Co., Ltd. The concentration of extracted DNA determined with the Qubit dsDNA HS Assay Kit in conjunction with the Qubit 4.0 Fluorometer. To amplify the bacterial 16S rRNA gene spanning the V3-V4 region, the primers used were 338F: 5'-ACTCCTACGGGAGGCAGCA-3' and 806R: 5'-GGACTACHVGGGTWTCTAAT-3'. The PCR commenced with an initial denaturation at 95 °C for 5 min, followed by 25 cycles that comprising a 30 s denaturation at 95 °C, annealed at 50 °C, and extended at 72 °C for 40 s. The final extension step was performed at 72 °C for 7 min. Purification of the PCR products carried out using Agencourt AMPure XP Beads (Beckman Coulter, Indianapolis, IN), and quantification was performed using the Qubit dsDNA HS Assay Kit in tandem with the Qubit 4.0 Fluorometer (Invitrogen, Thermo Fisher Scientific, Oregon, USA). After quantification, amplicons were combined in equal amounts. The resultant library was subjected to sequencing on the Illumina NovaSeq 6000 platform (Illumina, Santiago, CA, USA). The raw sequence data were then deposited in the NCBI Sequence Read Archive (SRA) database under accession number PRJNA1190609.

Table S1 The relative abundances at genus in the five groups

Genus	CK	PS0505	PS0510	PS1005	PS1010
unclassified_Gemmatimonadaceae	7.10 ± 0.34ab	7.95 ± 0.60ab	7.87 ± 0.29ab	6.26 ± 0.31b	9.67 ± 2.26a
unclassified_Bacteria	6.43 ± 0.74ab	7.13 ± 0.22a	7.00 ± 0.57a	6.98 ± 0.33a	6.02 ± 0.67b
unclassified_Vicinamibacterales	6.21 ± 0.55ab	6.66 ± 1.69ab	6.36 ± 1.12ab	5.77 ± 0.87b	7.75 ± 1.02a
unclassified_Vicinamibacteraceae	5.02 ± 0.31ab	5.54 ± 1.2ab	5.20 ± 0.70ab	4.94 ± 0.34b	6.16 ± 0.67a
unclassified_Chloroflexi	2.93 ± 0.37a	2.83 ± 0.47a	2.91 ± 0.66a	2.89 ± 0.13a	1.95 ± 0.37b
MND1	3.63 ± 0.47a	3.06 ± 0.37a	2.20 ± 0.41b	1.98 ± 0.17b	2.20 ± 0.34b
unclassified_Gemmatimonadota	1.94 ± 0.28ab	2.28 ± 0.16a	2.07 ± 0.17ab	1.65 ± 0.12b	2.11 ± 0.69ab
unclassified_Acidimicrobiia	1.92 ± 0.17b	1.86 ± 0.24b	2.20 ± 0.56ab	2.73 ± 0.46a	0.94 ± 0.30c
uncultured_gamma_proteobacterium	1.55 ± 0.28ab	1.46 ± 0.10b	1.35 ± 0.08b	1.65 ± 0.07ab	2.18 ± 0.77a
unclassified_Micrococcaceae	1.79 ± 0.18ab	1.45 ± 0.3ab	1.97 ± 0.38a	1.55 ± 0.39ab	1.32 ± 0.26b

Table S2 The relative abundances at phylum in the five groups

Phylum	CK	PS0505	PS0510	PS1005	PS1010
Proteobacteria	23.69 ± 0.64a	21.87 ± 1.28ab	20.16 ± 0.99b	23.27 ± 0.61a	19.67 ± 2.88b
Acidobacteriota	19.75 ± 1.58ab	21.22 ± 4.04ab	19.73 ± 2.33ab	17.51 ± 1.60b	22.09 ± 2.77a
Actinobacteriota	14.05 ± 0.19a	14.03 ± 3.52a	17.07 ± 3.72a	17.35 ± 1.46a	13.53 ± 1.26a
Gemmatimonadota	11.21 ± 0.79bc	12.44 ± 0.46ab	12.05 ± 0.19abc	9.33 ± 0.51c	14.06 ± 3.56a
Chloroflexi	7.58 ± 0.35ab	7.09 ± 1.23b	8.35 ± 0.70ab	8.55 ± 0.37a	7.04 ± 0.90b
unclassified_Bacteria	6.43 ± 0.74ab	7.13 ± 0.22a	7.00 ± 0.57a	6.98 ± 0.33a	6.02 ± 0.67b

Myxococcota	4.93 ± 0.20ab	4.43 ± 0.22bc	3.95 ± 0.26c	4.33 ± 0.30bc	5.13 ± 0.78a
Bacteroidota	4.07 ± 0.04a	4.37 ± 0.52a	4.18 ± 0.24a	4.13 ± 0.44a	4.87 ± 2.10a
Patescibacteria	2.20 ± 0.24b	2.78 ± 0.29ab	2.44 ± 0.25ab	3.28 ± 0.57a	2.69 ± 1.12ab
Methylomirabilota	1.15 ± 0.10a	0.96 ± 0.10a	1.10 ± 0.39a	0.89 ± 0.09a	1.17 ± 0.41a

Table S3 The relative abundances at family in the five groups

Family	CK	PS0505	PS0510	PS1005	PS1010
Gemmatimonadaceae	8.11 ± 0.46b	8.87 ± 0.59ab	8.94 ± 0.25ab	6.90 ± 0.40b	10.99 ± 2.76a
unclassified_Vicinamibacterales	7.38 ± 0.76b	8.05 ± 1.86ab	7.56 ± 1.25b	6.77 ± 0.89b	9.73 ± 1.00a
unclassified_Bacteria	6.43 ± 0.74ab	7.13 ± 0.22a	7.00 ± 0.57a	6.98 ± 0.33a	6.02 ± 0.67b
Vicinamibacteraceae	5.70 ± 0.39a	6.18 ± 1.36a	5.81 ± 0.71a	5.56 ± 0.39a	6.32 ± 0.74a
Nitrosomonadaceae	5.07 ± 0.80a	4.48 ± 0.35a	2.94 ± 0.33b	2.62 ± 0.19b	3.41 ± 0.65b
unclassified_Chloroflexi	2.93 ± 0.37a	2.83 ± 0.47a	2.91 ± 0.66a	2.89 ± 0.13a	1.95 ± 0.37b
Sphingomonadaceae	2.43 ± 0.15a	2.12 ± 0.14ab	1.97 ± 0.23b	1.85 ± 0.16b	2.01 ± 0.26b
unclassified_Gemmatimonadota	1.94 ± 0.28a	2.28 ± 0.16ab	2.07 ± 0.17ab	1.65 ± 0.12b	2.11 ± 0.69ab
Xanthomonadaceae	2.69 ± 0.11a	2.15 ± 0.13b	1.91 ± 0.35b	1.93 ± 0.34b	0.65 ± 0.17c
Micrococcaceae	1.81 ± 0.19ab	1.45 ± 0.30b	2.19 ± 0.52a	1.60 ± 0.42ab	1.39 ± 0.26b

Table S4 Major topological properties of empirical MENs microbial communities in the five groups

Network Indexes	CK	PS0505	PS0510	PS1005	PS1010
Total nodes	370	303	332	405	167
Total links	2816	2116	2246	3679	503
R square of power-law	0.219	0.188	0.219	0.186	0.437
PP	94.39%	81.66%	90.74%	92.12%	76.54%
NP	5.61%	18.34%	9.26%	7.88%	23.46%
Average degree (avgK)	15.222	13.967	13.530	18.168	6.024
Average clustering coefficient (avgCC)	0.651	0.608	0.618	0.667	0.528
Average path distance (GD)	4.941	5.105	5.229	4.558	5.464
Geodesic efficiency (E)	0.288	0.278	0.284	0.299	0.269
Harmonic geodesic distance (HD)	3.475	3.591	3.519	3.344	3.722
Maximal degree	37	34	33	44	16
Nodes with max degree	ASV473	ASV115	ASV130	ASV608	ASV25932
Centralization of degree (CD)	0.059	0.067	0.059	0.064	0.061
Maximal betweenness	7663.329	6534.587	8373.025	7558.951	1664
Nodes with max betweenness	ASV1059	ASV94	ASV248	ASV524	ASV38
Centralization of betweenness (CB)	0.107	0.134	0.146	0.088	0.108
Maximal stress centrality	1562710	5420014	3509934	6571176	96536
Nodes with max stress centrality	ASV1059	ASV94	ASV248	ASV524	ASV38
Centralization of stress centrality (CS)	22.307	114.569	61.786	78.792	6.445
Maximal eigenvector centrality	0.211	0.210	0.175	0.177	0.299

Nodes with max eigenvector centrality	ASV473	ASV115	ASV226	ASV608	ASV25932
Centralization of eigenvector centrality (CE)	0.192	0.184	0.149	0.159	0.270
Density (D)	0.041	0.046	0.041	0.045	0.036
Reciprocity	1	1	1	1	1
Transitivity (Trans)	0.715	0.621	0.644	0.727	0.604
Connectedness (Con)	0.537	0.752	0.615	0.590	0.535
Efficiency	0.928	0.942	0.938	0.928	0.942
Hierarchy	0	0	0	0	0
Lubness	1	1	1	1	1

Table S5 The relative abundance of key functional genes about N cycle in different microbial communities

Genes for proteins/enzyzmes	Functional	CK	PS0505	PS0510	PS1005	PS1010
psbABCDEF	Photosystem II	2.47E-06a	2.57E-06a	1.79E-06a	1.08E-06a	1.24E-06a
psaABCDEF	Photosystem I	1.46E-06a	1.30E-06a	9.15E-07a	6.02E-07a	6.26E-07a
petABCDGLMN	Cytochrome b6/f complex	5.83E-06a	6.22E-06a	5.65E-06a	5.24E-06a	3.94E-06b
pufM, pufL	Anoxygenic photosystem II	5.27E-06b	4.36E-06b	4.37E-06b	7.64E-06a	5.54E-06b
RuBisCo	RuBisCo	5.57E-05a	5.22E-05ab	4.49E-05ab	4.60E-05ab	3.99E-05b
prkB	CBB cycle	4.93E-05a	5.02E-05a	4.58E-05a	4.85E-05a	4.62E-05a
aclAB, ccsAB	rTCA cycle	6.87E-06a	5.94E-06a	6.60E-06a	6.17E-06a	5.71E-06a
acsABCDE	Wood-Ljungdahl pathway	2.57E-06b	3.34E-06ab	3.93E-06a	3.43E-06ab	1.14E-06c
3-Hydroxypropionate	3-Hydroxypropionate Bicycle	1.44E-05ab	1.31E-05b	1.51E-05ab	1.61E-05a	1.37E-05ab
glk, pfk, pyk	Glycolysis	9.35E-04b	9.41E-04b	9.66E-04a	9.45E-04ab	9.58E-04ab
	Entner-Doudoroff pathway, glucose-6P -> glyceraldehyde-3P + pyruvate	3.59E-04b	3.56E-04b	3.64E-04ab	3.80E-04a	3.71E-04ab
fbp, pck	Gluconeogenesis	4.56E-04a	4.53E-04a	4.57E-04a	4.57E-04a	4.32E-04b
	TCA cycle	1.00E-03ab	1.01E-03a	1.01E-03ab	9.86E-04b	1.02E-03a
mcrABG	Methanogenesis	9.49E-07b	4.57E-07c	1.19E-06ab	1.57E-06a	1.49E-07c
mtaABC	Methanogenesis, methanol -> methane	7.80E-09a	1.52E-08a	1.16E-09a	1.43E-08a	1.68E-08a
cdhCDE	Methanogenesis, acetate -> methane	1.77E-06b	2.36E-06ab	2.59E-06a	2.26E-06ab	9.08E-07c
	Methanogenesis, CO2 -> methane	8.92E-06bc	8.83E-06bc	1.26E-05b	2.08E-05a	5.10E-06c
mmoBCDXYZ, amoABC	Methane oxidation, methane -> methanol	1.65E-06b	1.37E-06b	1.32E-06b	1.45E-06b	8.90E-06a

mxaFI, xoxF	Methane oxidation, methanol -> formaldehyde	1.22E-05bc	9.86E-06c	1.91E-05b	3.27E-05a	5.30E-06c
LDH	Fermentation to lactate, pyruvate -> lactate	1.65E-05a	1.72E-05a	1.71E-05a	1.86E-05a	2.00E-05a
pf1D	Fermentation to formate, pyruvate -> formate	1.56E-05a	1.62E-05a	1.55E-05a	1.85E-05a	1.67E-05a
fdh	Fermentation to formate -> CO2 & H2	1.82E-04a	1.81E-04a	1.81E-04a	1.82E-04a	1.75E-04a
poxB, poxL, acyP	Fermentation to acetate, pyruvate -> acetate	4.07E-04c	4.07E-04c	4.20E-04b	3.98E-04c	4.50E-04a
ach1, eutD, pta, acyP	Fermentation to acetate, acetyl-CoA -> acetate	3.84E-04b	3.85E-04b	3.92E-04ab	3.65E-04c	3.99E-04a
	Fermentation to acetate, lactate -> acetate	1.24E-05a	1.30E-05a	1.01E-05a	1.13E-05a	1.56E-05a
ald	Fermentation to ethanol, acetate to acetylaldehyde	1.27E-03a	1.27E-03a	1.32E-03ab	1.29E-03bc	1.33E-03a
	Fermentation to ethanol, acetyl-CoA to acetylaldehyde	6.55E-05b	6.56E-05b	6.75E-05b	7.82E-05ab	8.60E-05a
adh, mdh	Fermentation to ethanol, acetylaldehyde to ethanol	9.00E-04bc	8.65E-04c	9.23E-04b	9.14E-04b	1.00E-03a
	Fermentation to succinate (phosphoenolpyruvate to succinate via oxaloacetate, malate & fumarate)	6.59E-04b	6.55E-04b	6.49E-04b	6.39E-04a	6.56E-04b

Table S6 The relative abundance of key functional genes about N cycle in different microbial communities

**Genes for proteins/enzyzmes**	**Functional**	**CK**	**PS0505**	**PS0510**	**PS1005**	**PS1010**
NarGHI, napAB	Dissimilatory nitrate reduction, nitrate -> nitrite	6.28E-05a	5.81E-05b	5.35E-05c	5.59E-05bc	5.57E-05bc
nirBD, nrfAH	Dissimilatory nitrate reduction, nitrite -> ammonia	2.56E-04bc	2.55E-04bc	2.53E-04c	2.71E-04a	2.67E-04ab
narB, nasAB	Assimilatory nitrate reduction, nitrate -> nitrite	1.04E-04ab	1.01E-04b	1.08E-04ab	1.13E-04a	1.08E-04ab
NIT-6, nirA	Assimilatory nitrate reduction, nitrite -> ammonia	1.07E-05a	1.06E-05a	1.12E-05a	8.86E-06a	1.10E-05a
nirK, nirS	Denitrification, nitrite -> nitric oxide	7.89E-05a	7.92E-05a	7.13E-05ab	6.84E-05ab	5.83E-05b
norBC	Denitrification, nitric oxide -> nitrous oxide	2.60E-05a	2.27E-05a	2.43E-05a	2.31E-05a	1.21E-05b
nosZ	Denitrification, nitrous oxide -> nitrogen	4.75E-05ab	4.93E-05a	4.93E-05a	4.53E-05ab	3.74E-05b
nifKDH	Nitrogen fixation, nitrogen -> ammonia	6.22E-06b	6.18E-06b	7.81E-06a	6.72E-06ab	4.06E-06c

amoABC	Nitrification, ammonia -> hydroxylamine	1.60E-06b	1.23E-06b	1.13E-06b	1.25E-06b	8.68E-06a
hao	Nitrification, hydroxylamine -> nitrite	9.00E-07b	6.91E-07b	6.25E-07b	7.41E-07b	6.32E-06a
nxrAB	Nitrification, nitrite -> nitrate	5.88E-05a	5.34E-05b	4.86E-05b	5.19E-05b	5.36E-05ab

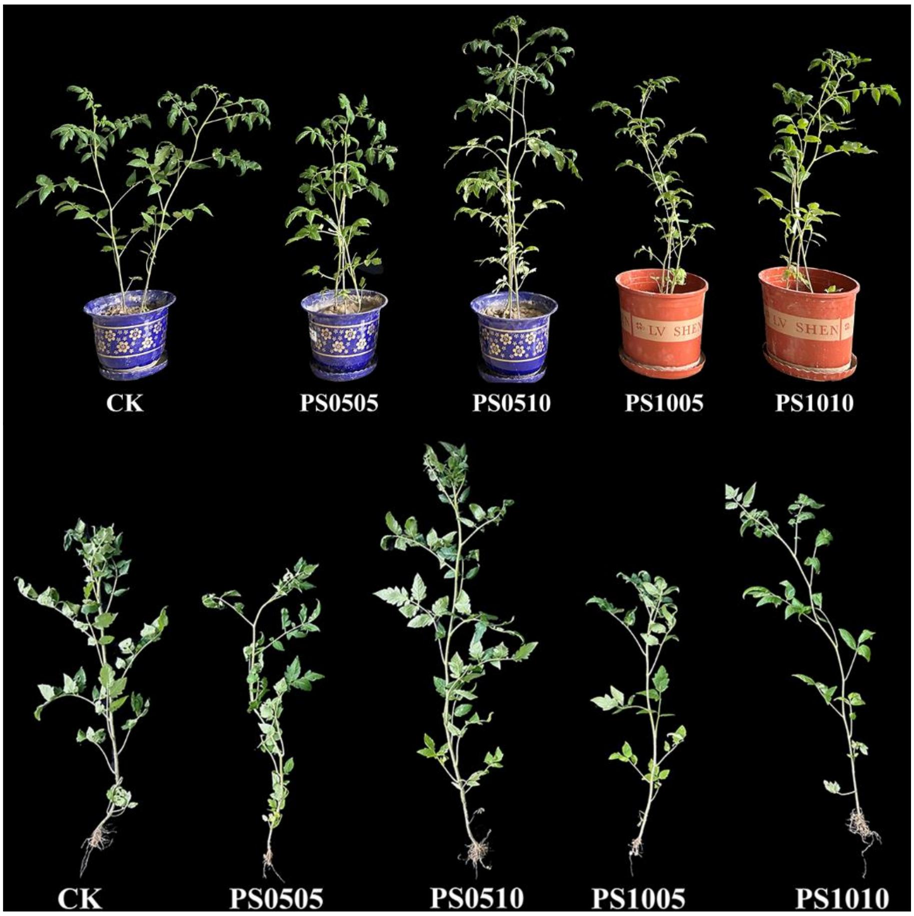

Figure S1 The harvested *Solanum lycopersicum* L. after 80 days

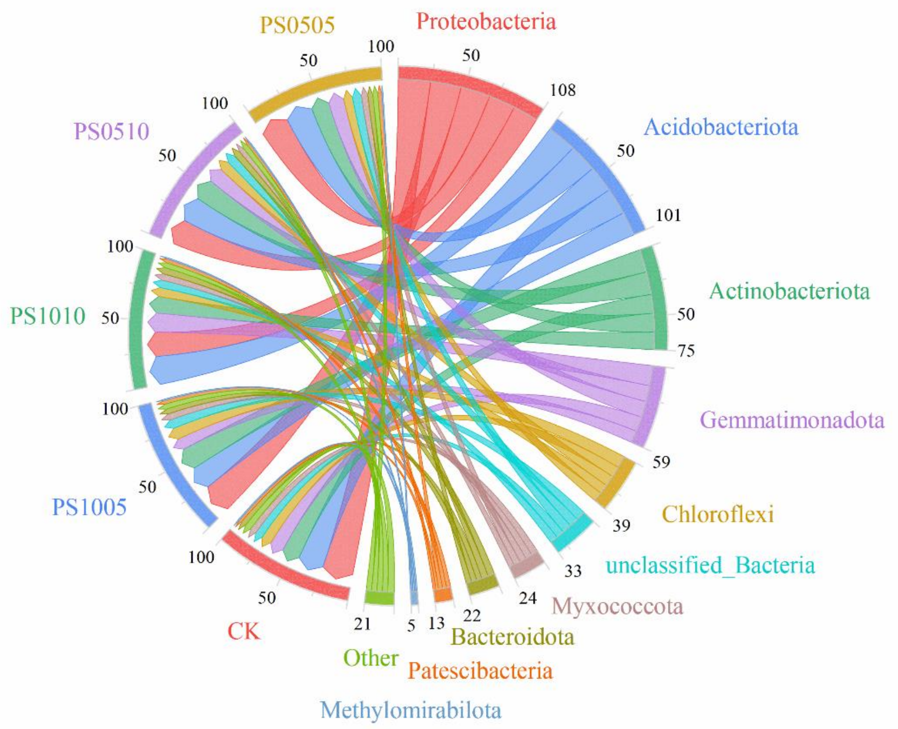

Figure S2 The abundance of microorganisms at the phylum level

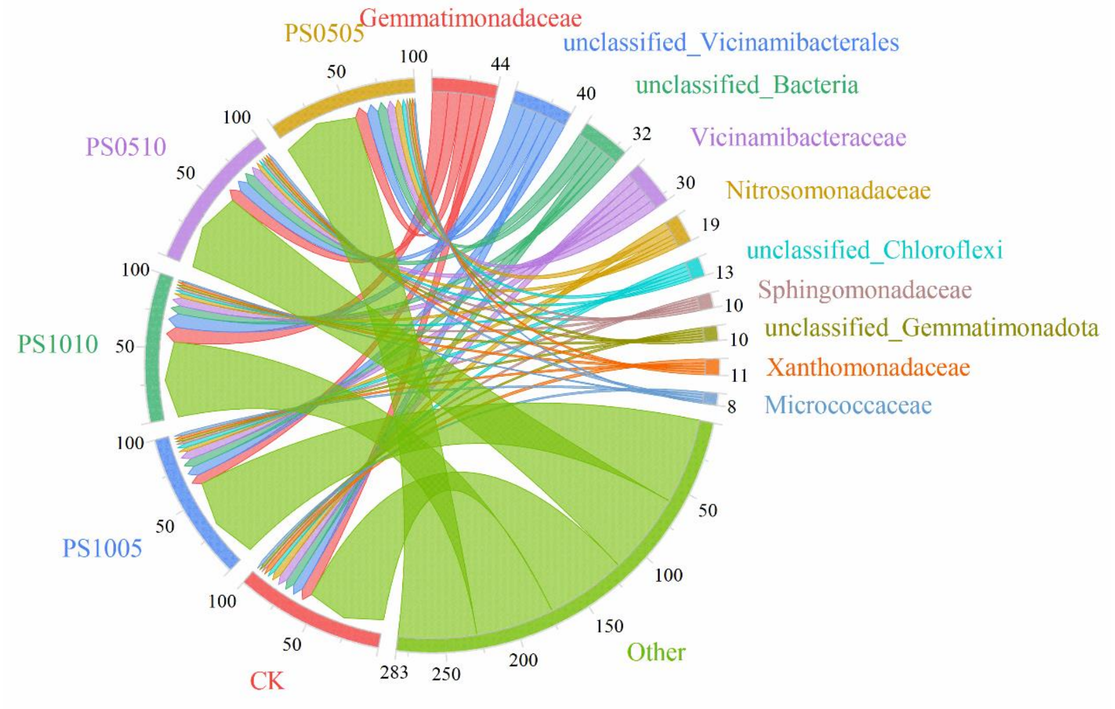

Figure S3 The abundance of microorganisms at the family level

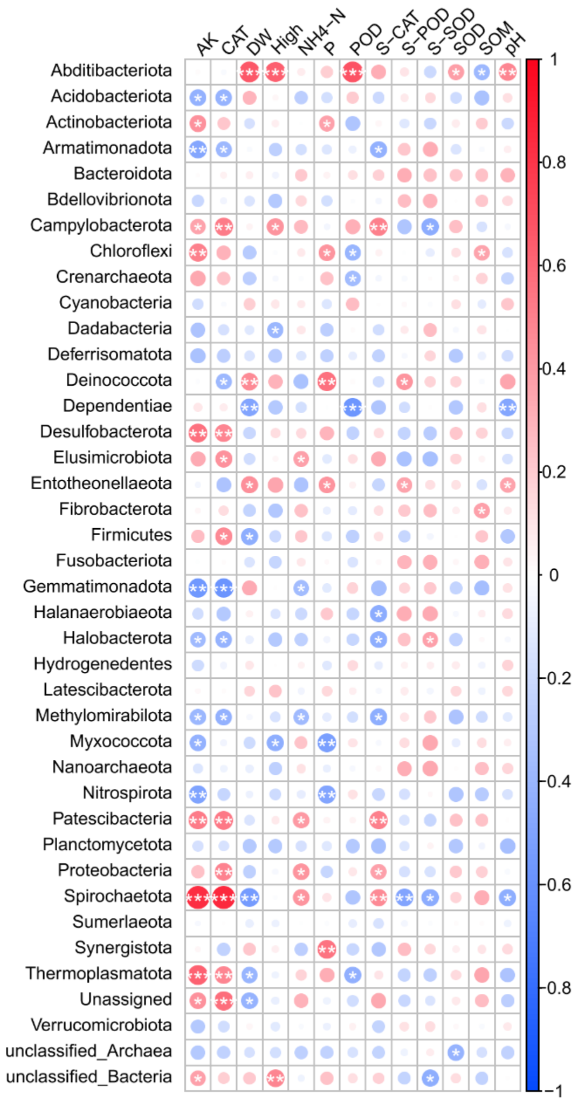

Figure S4 Spearman correlations of phyla, soil and *Solanum lycopersicum* L. parameters

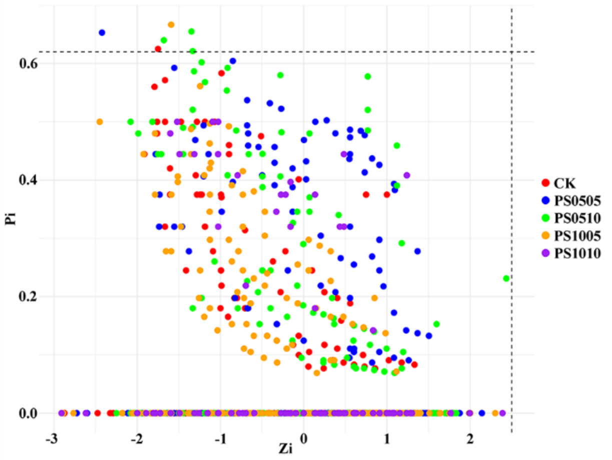

Figure S5 ZP diagram
